# Modulation of adipose inflammation by cellular retinoic acid-binding protein 1

**DOI:** 10.1038/s41366-022-01175-3

**Published:** 2022-07-06

**Authors:** Chin-Wen Wei, Jennifer Nhieu, Yu-Lung Lin, Li-Na Wei

**Affiliations:** Department of Pharmacology, University of Minnesota Medical School, Minneapolis, MN 55455 USA

**Keywords:** Cell biology, Physiology

## Abstract

**Objectives:**

Obesity, a metabolic syndrome, is known to be related to inflammation, especially adipose tissue inflammation. Cellular interactions within the expanded white adipose tissue (WAT) in obesity contribute to inflammation and studies have suggested that inflammation is triggered by inflamed adipocytes that recruit M1 macrophages into WAT. What causes accumulation of unhealthy adipocytes is an important topic of investigation. This study aims to understand the action of Cellular Retinoic Acid Binding Protein 1 (CRABP1) in WAT inflammation.

**Methods:**

Eight weeks-old wild type (WT) and *Crabp1* knockout (CKO) mice were fed with a normal diet (ND) or high-fat diet (HFD) for 8 weeks. Body weight and food intake were monitored. WATs and serum were collected for cellular and molecular analyses to determine affected signaling pathways. In cell culture studies, primary adipocyte differentiation and bone marrow-derived macrophages (BMDM) were used to examine adipocytes’ effects, mediated by CRABP1, in macrophage polarization. The 3T3L1-adipocyte was used to validate relevant signaling pathways.

**Results:**

CKO mice developed an obese phenotype, more severely under high-fat diet (HFD) feeding. Further, CKO’s WAT exhibited a more severe inflammatory state as compared to wild type (WT) WAT, with a significantly expanded M1-like macrophage population. However, this was not caused by intrinsic defects of CKO macrophages. Rather, CKO adipocytes produced a significantly reduced level of adiponectin and had significantly lowered mitochondrial DNA content. CKO adipocyte-conditioned medium, compared to WT control, inhibited M2-like (CD206^+^) macrophage polarization. Mechanistically, defects in CKO adipocytes involved the ERK1/2 signaling pathway that could be modulated by CRABP1.

**Conclusions:**

This study shows that CRABP1 plays a protective role against HFD-induced WAT inflammation through, in part, its regulation of adiponectin production and mitochondrial homeostasis in adipocytes, thereby modulating macrophage polarization in WAT to control its inflammatory potential.

## Introduction

Obesity is an important component of metabolic syndromes and can increase the risk of numerous diseases such as cardiovascular diseases, type 2 diabetes mellitus, and other inflammatory diseases, etc. It is characterized by the expansion of white adipose tissue (WAT), caused by both hyperplasia and hypertrophy of adipocytes [1, 2]. Abnormal adipocyte hypertrophy results in changes in the secretary patterns of adipocyte cytokines and adipokines. For example, overproduction of inflammatory cytokines including IL-6, TNFα and MCP1, and dysregulation in several adipokines like leptin and adiponectin can first induce low-grade inflammation and insulin resistance. Without intervention, this usually proceeds to a state of chronic inflammation accompanied by infiltration of inflammatory immune cells such as M1 macrophages, B, and Th1 cells, further enhancing the production of inflammatory cytokines [3, 4].

Adipose tissue homeostasis is regulated by multiple factors. The health of adipocytes and proper production/secretion of adipokines are critical to its physiological function. Adiponectin is the most abundant peptide hormone produced and secreted by adipocytes and especially plays an important role in the communication of adipose tissues with other organ systems to regulate energy homeostasis, metabolism and inflammation. Reduction in adiponectin production often results in obesity-related diseases especially elevated inflammation [5]. As such, regulation of adiponectin production/secretion, as well as its downstream signaling, is critical to health. To this end, adiponectin gene transcription is known to be inhibited by inflammatory cytokines via JNK or ERK1/2 pathways, and also suppressed by PKA-mediated CREB activation; whereas insulin can positively regulate adiponectin by PI3K/AKT pathway [6–9]. Uncovering new pathways/factors that can regulate adiponectin production/secretion as well as its signaling pathway in a physiological context is an important topic in understanding and managing obesity-related diseases.

An additional potential defect in adipocytes is the alteration/damage in their mitochondria. Inflammatory process increases the production of reactive oxygen species (ROS) that causes oxidative stress and impairs mitochondrial biogenesis/function. This also causes tissue inflammation and pathogenesis of obesity [10–12]. Multiple human studies have demonstrated mitochondrial defects in adipocytes of people with obesity [13–16]. Numerous studies have attempted to understand the molecular mechanisms underlying adipocyte dysfunction; one mechanism involves the ERK1/2 signaling pathway which contributes to adipose tissue inflammatory response [17], promotes adipocyte differentiation and hypertrophy [18, 19] and is also associated with mitochondria-mediated death [20]. Therefore, it is generally believed that a well-controlled ERK1/2 signaling pathway in adipocytes is important for the maintenance of healthy adipose tissue. Identifying factors modulating this signaling pathway in adipocytes is important for understanding the mechanisms regulating the health of adipose tissues.

Cellular Retinoic Acid Binding Protein 1 (CRABP1) is a cytoplasmic protein with a high affinity toward retinoic acid (RA) [21]. Besides binding RA, CRABP1 is known to modulate at least two cytosolic signaling, MAPK and CaMKII, pathways in a cell context-dependent manner [22]. While its functional roles in several cell types have been uncovered recently, including neuron differentiation [23], neuronal exosome secretion [24], endocrine homeostasis [25], and cardiomyocyte function [26], how CRABP1 functions specifically in adipocytes remain elusive. Importantly, we have found that *Crabp1* gene expression is tightly regulated during adipocyte differentiation, through hormonal induction followed by epigenetic silencing [27]. Interestingly, *Crabp1* knockout (CKO) mice, as compared to WT, had elevated plasma glucose levels and apparently more severe adipocyte hypertrophy under a high-fat diet (HFD) feeding. This suggests that CRABP1 probably plays a protective role against the development of obesity through, at least partially, regulating adipocytes and adipose tissue inflammation [28]. This study aims to specifically examine the action of CRABP1 in adipose tissue inflammation to understand the cellular and molecular basis of WAT inflammation.

## Method

### Animals

Male mice were used for all experiments. Wild type (WT) C57BL/6 mice were obtained from The Jackson Laboratory and housed in the University of Minnesota animal facilities. CKO mice were maintained as previously described [28, 29]. Briefly, C57BL/6J background CKO mice were generated from a *Crabp1*-targeted DE3 (ES) clone containing a 5 bp Not1 insertion in exon 1 (the fifth codon of the *Crabp1* coding region) to ablate *Crabp1* expression. 8-weeks-old WT and CKO mice were fed with normal diet (ND; F4031 Control Diet, Bio-Serv) or high-fat diet (HFD; F3282; Bio-Serv; 60% calories from fat) for 8 weeks. All Experimental procedures were conducted following the NIH guidelines and the protocols approved by the University of Minnesota Institutional Animal Care and Use Committee.

### White adipose tissue isolation

Mice were euthanized by CO_2_ asphyxiation. White adipose tissue (WAT) from subcutaneous and visceral fat were harvested from mice, and digested with 10 mM  CaCl_2_, 3 mg/ml Collagenase II (Worthington Biochemical #LS004177), and 0.4 mg/mL DNase I (Roche #10104159001), and FACS buffer (1 mM EDTA, 5% FBS in PBS) at 37 °C for 30–40 min. Cell suspensions were strained through a 100 μm nylon mesh filter and centrifuged at 300 × *g* for 5 min at 4 °C. The supernatant was decanted, and red blood cells were removed with ACK buffer (NH_4_Cl 0.15 M, KHCO_3_ 10 mM, EDTA 0.1 mM) at room temperature for 2 min. Cells were then resuspended in FACS buffer. Single-cell suspensions were counted using trypan blue staining.

### Flow cytometry

Single-cell suspensions were prepared from WAT. Cells were first incubated with anti-mouse CD16/32 antibody to prevent non-specific binding (BD Biosciences, clone: 2.4G2). Then cells were stained with fluorescence-conjugated antibodies (Supplemental Experimental Methods and Materials) in FACS buffer, washed, and resuspended in FACS buffer with propidium iodide (Sigma-Aldrich P4684). Flow cytometry experiments were performed on BD LSR II (BD Biosciences) and analyzed using FlowJo software.

### In vitro and ex vivo adipocyte differentiation

Single-cell suspensions were prepared from WAT and cultured in completed F12/DMEM medium (Thermo Fisher Scientific #11320033) containing 100 U/mL penicillin, 100 mg/mL streptomycin (Gibco), and 10% fetal bovine serum (FBS) for 2 h. Nonadherent cells were removed, and the remaining adherent cells were cultured for 2–3 days. When the cell density reached 80%, the preadipocytes were counted and seeded in 6-well plate and left overnight. Then differentiation cocktail (0.5 mM isobutylmethylxanthine (IBMX) (Sigma-Aldrich #I7018), 1 μM dexamethasone (Sigma-Aldrich #D4902)) in completed F12/DMEM medium was added and incubated for 2 days. Then medium was changed to 10 μg/ml insulin (Sigma-Aldrich #I6634) in completed F12/DMEM medium for 4–6 days.

The 3T3L1 cell line was a kind gift from Dr. Xiaoli Chen at the University of Minnesota. Cells were regularly tested for mycoplasma contamination. Adipocyte differentiation of 3T3L1 cells was performed by incubating cells with the same differentiation cocktail as above. After day 2, cells were maintained in completed F12/DMEM containing insulin until day 8, and then treated with 25 µM PD98059 or DMSO control for 24 h. For *Crabp1* silencing experiments, 3T3L1 cells were transfected with negative-control scramble siRNA or *Crabp1* siRNA using Hiperfect transfection reagent (Qiagen) according to manufacturer instructions. The siRNAs were transfected prior to differentiation and on day 4 after differentiation. Mouse *Crabp1* siRNA sequences were 5’ CACGTGGGAGAATGAGAACAA 3’ and 5’ CAGCTTGTTCCTGCTTCATGA 3’.

### Statistical analysis

Sample size for animal experiments was determined based on previous studies of HFD mouse model. Detail sample size was chosen for in vivo experiments: Body weight, *n* = 11 per group. Serum adiponectin level, *n* = 5–7 mice per group. Flow cytometry analysis, *n* = 5–10 mice per group. No animals were excluded from the analyses. When comparing results from four groups under two conditions (ND or HFD), two-way ANOVA was used. Two-tailed Student’s *t* test was used when appropriate for comparison among the groups. Data were normally distributed and variance was similar among groups that were being statistically compared. Data were presented as means ± SEM. The comparison was considered statistically significant when *p* values ≤ 0.05 (*p < 0.05; **p < 0.01; ***p < 0.001). Prism 6.0 (GraphPad) was used for plotting data and statistical analysis.

## Results

### CRABP1 deficiency altered the distribution of polarized macrophages in WAT

**Fig. 1:**
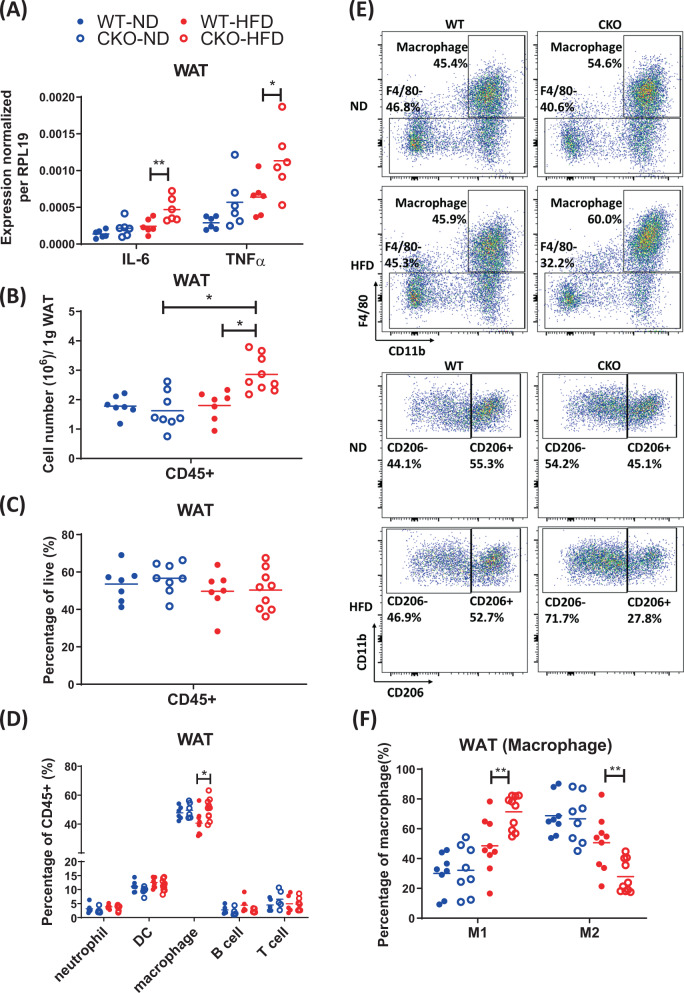
Crabp1 KO (CKO) mice exhibit an inflammatory phenotype in WAT under HFD feeding. Adult (8-week-old) WT and CKO mice were fed a normal diet (ND) or high-fat diet (HFD) for 8 weeks. **A** Transcription of *IL-6* and *TNFα* in white adipose tissue (WAT) was analyzed by qPCR and normalized to *RPL19* as an internal control. **B**–**F** Visceral WAT from WT and CKO mice fed a ND or HFD was analyzed by flow cytometry. Cells were gated based on single cells and live cells (propidium iodide^−^). **B**, **C** Cell number and percentage of CD45^+^ leukocytes. **D** Percentage of CD45^+^Gr-1^+^ (neutrophils), F4/80^-^CD11c^+^ (dendritic cells; DC), CD45^+^CD11b^+^ F4/80^+^ (macrophages), CD45^+^B220^+^ (B cells), and CD45^+^TCRb^+^ (T cells). **E** Profiles of macrophage subpopulations in WAT. The total macrophage population was further gated into CD206^−^ (M1) and CD206^+^(M2) subpopulations. **F** Percentages of M1 and M2 macrophages in WAT. Results were obtained from two to four independent experiments for statistical analysis using two-way ANOVA, *p < 0.05, **p < 0.01.

CKO mice, under HFD feeding, developed extreme obesity as compared to WT mice. We have previously documented fat accumulation and adipocyte hypertrophy in visceral WAT of CKO mice [28]. In this current study, we aimed to determine the molecular and cellular basis of abnormal WAT in HFD-fed CKO mice. We first found significantly higher expression of *IL-6* and *TNFα* in HFD-fed CKO, as compared to HFD-fed WT mice (Fig. 1A). We then compared the immune cell populations within the WAT between WT and CKO mice. To avoid the potential effects of HFD on the development of the immune system, we fed adult (8-weeks-old) WT and CKO mice with ND or HFD for 8 weeks, then monitored the phenotype of CKO mice under ND and HFD feedings for comparison to WT mice. We found that CKO mice gained more body weight and WAT mass under HFD feeding (Supplementary Fig. S1). We next examined immune cell populations within the WAT. As shown, although the number of total leukocytes (CD45^+^) was not affected in WT mice between ND and HFD group, the number of total leukocytes in CKO mice was increased under HFD feeding, suggesting inflammation in CKO’s WAT (Fig. 1B). We carefully examined the percentage of total leukocytes in WAT, and found no significant difference between WT and CKO mice (Fig. 1C). However, the percentage of myeloid cell populations was altered in CKO, with a significantly increased macrophage population under HFD feeding (Fig. 1D, E; upper panel). In carefully examining macrophage subpopulations, we found that the ratio of M1 and M2 macrophages was apparently different between WT and CKO that, the percentage of CD206^+^ population (M2-like macrophage) was much lower in CKO mice under HFD feeding (Fig. 1E; bottom panel and 1F), indicative of elevated WAT inflammation in CKO. This result showed preferential M1-like macrophage polarization as one feature of WAT inflammation in CKO. This could be due to CRABP1 deficiency that affected resident macrophage polarization, or the recruitment of inflammatory macrophages into WAT.

### CRABP1 does not affect intrinsic polarization potential in macrophage

**Fig. 2:**
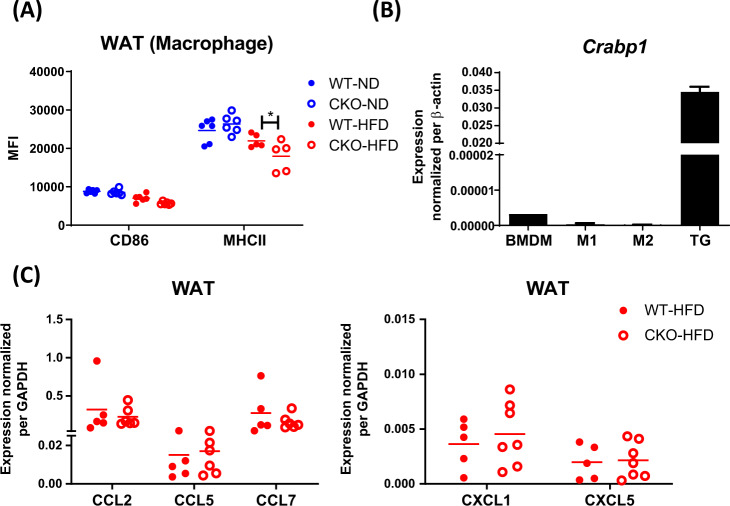
CRABP1 is dispensable for macrophage migration and intrinsic ability in polarization. **A** Adult (8-week-old WT and CKO) mice were fed a ND or HFD for 8 weeks. Plot diagrams show MHCII and CD86 mean fluorescence intensity (MFI) values in the macrophages of WAT. **B** Bone marrow-derived macrophages (BMDM) from WT were differentiated into M1 via LPS and IFNγ treatment, and into M2 via IL-4 treatment. qPCR of *Crabp1* expression in WT BMDM, M1 and M2 macrophages, and WT thyroid gland tissue. *β-actin* was used as an internal control. Error bars are shown as means ± SEM. **C** *CCL2, CCL5, CCL7, CXCL1* and *CXCL5* transcripts in WAT of HFD-fed WT and CKO mice were analyzed by qPCR and normalized to *GAPDH* as an internal control. Results were obtained from two independent experiments for statistical analysis using two-way ANOVA. *p < 0.05.

We next confirmed the abnormality in WAT macrophages of CKO mice by examining the surface expression of MHCII and CD86 in macrophages of WAT. The data showed that macrophages from CKO’s WAT had a slightly lower, but not statistically significant, mean fluorescence intensity (MFI) of CD86, and significantly lower MFI of MHCII as compared to WT under HFD feeding (Fig. 2A). This result confirmed the abnormalities in macrophage populations in CKO’s WAT. We first suspected a role for CRABP1 in the intrinsic property of macrophage lineage. Upon examining the expression of *Crabp1* in bone marrow-derived macrophages (BMDM), we found negligible expression of *Crabp1* mRNA, less than 10000-fold as compared to *Crabp1* expression in thyroid tissue which was included as a positive control (Fig. 2B). We next examined whether CKO macrophages were defected in their polarization potential by comparing M1 (marked by MHCII and CD86) and M2 (marked by CD206) [30] populations of BMDM collected from WT and CKO mice fed a ND or HFD. There was no difference in M1 or M2 distribution between WT and CKO (Supplementary Fig. S2). Therefore, CRABP1 is not likely to affect macrophage’s intrinsic polarization potential. We next determined whether CRABP1 affected macrophage infiltration/recruitment from circulation by examining chemokine expression patterns in WAT. As shown in Fig. 2C, chemokine gene expression was not altered. These data showed that CRABP1 deficiency affected adipose tissue inflammation more likely through locally modulating resident macrophage polarization. Although it remains to be further examined whether macrophage infiltration was altered.

### CRABP1 affects adipocytes’ ability to regulate macrophage polarization

**Fig. 3:**
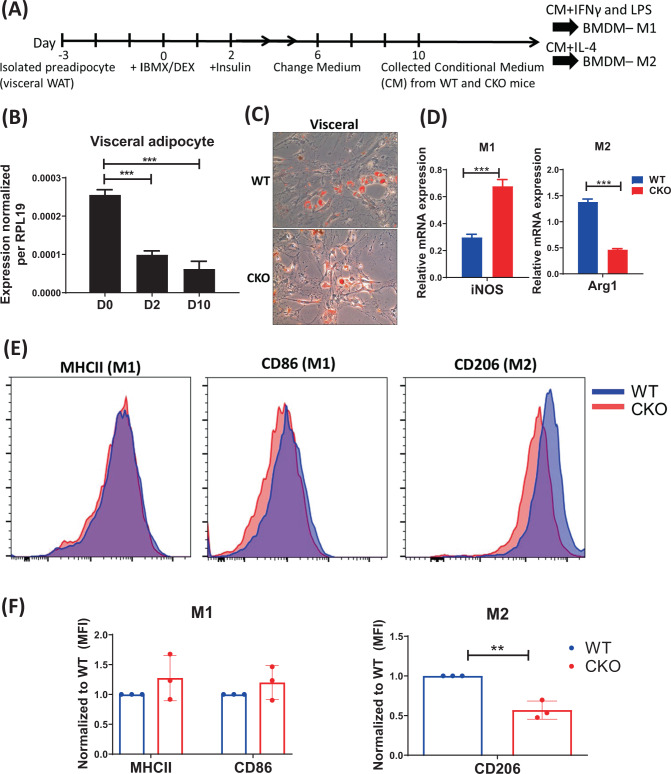
Conditioned media (CM) from visceral white adipocytes of CKO mice alters macrophage polarization by promoting M1 and inhibiting M2 macrophages. **A** Experimental diagram for detecting the effects of adipocyte’s conditioned media (CM). **B** *Crabp1* expression during WT visceral adipocyte differentiation was analyzed by qPCR and normalized to *RPL19*. **C** Representative Oil-O-Red staining of differentiated- visceral adipocytes from WT and CKO mice. **D**–**F** CM from visceral adipocytes of WT and CKO mice was collected and used to treat WT BMDM. LPS/ IFNγ or IL-4, in combination with CM, was used in M1 or M2 polarization, respectively. **D** qPCR analyses of *iNOS* and *Arg1* expression in M1 and M2 macrophages. **E** Representative flow cytometry histograms of MHCII (M1), CD86 (M1) and CD206 (M2) staining of M1 and M2 macrophages. **F** Quantification of MHCII, CD86, and CD206 staining. MFI values were normalized to WT. *n* = 3; qPCR data shown are means ± SEM of three independent experiments. Student’s *t* test, **p < 0.01, ***p < 0.001.

**Fig. 4:**
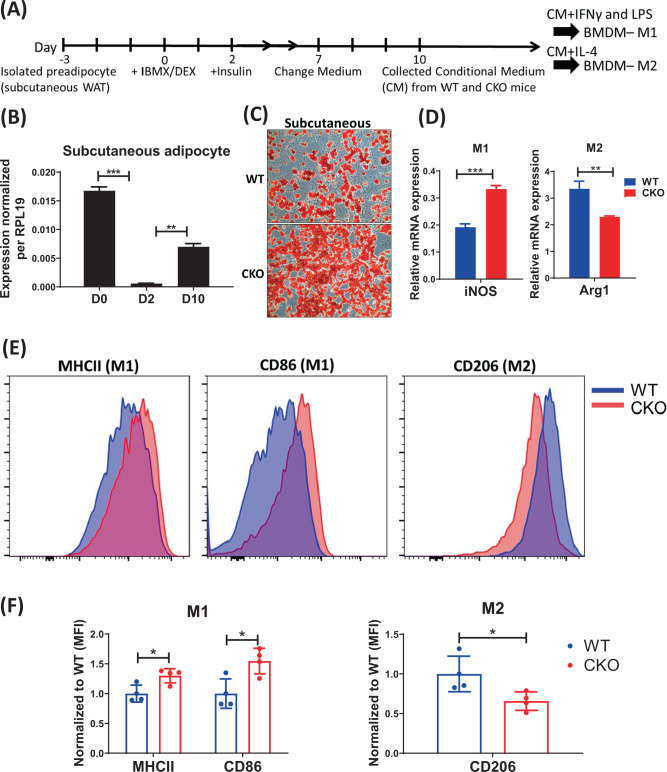
Conditioned media (CM) from subcutaneous white adipocytes of CKO mice alters macrophage polarization by promoting M1 and inhibiting M2 macrophages. **A** Experimental diagram for detecting the effects of adipocyte’s conditioned media (CM). **B** *Crabp1* expression during WT subcutaneous adipocyte differentiation was analyzed by qPCR and normalized to *RPL19*. **C** Representative Oil-O-Red staining of differentiated-subcutaneous adipocytes from WT and CKO mice. **D**–**F** CM from subcutaneous adipocytes of WT and CKO mice was collected and used to treat WT BMDM. LPS/ IFNγ or IL-4, in combination with CM, was used in M1 or M2 polarization, respectively. **D** qPCR analyses of *iNOS* and *Arg1* expression in M1 and M2 macrophages. **E** Representative flow cytometry histograms of MHCII (M1), CD86 (M1) and CD206 (M2) staining of M1 and M2 macrophages. **F** Quantification of MHCII, CD86, and CD206 staining. MFI values were normalized to WT. *n* = 4; qPCR data shown are means ± SEM of four independent experiments. Student’s *t* test, *p < 0.05, **p < 0.01, ***p < 0.001.

We next examined whether WAT adipokines were altered in CKO mice, which would have affected resident macrophage polarization in CKO’s WAT. To test this possibility, we cultured primary preadipocytes from visceral and subcutaneous WATs of these animals for further adipocyte differentiation (Figs. 3A and 4A). We then collected mature adipocyte-conditioned medium (CM) from these adipocytes to feed WT BMDM cultures which were induced for M1 and M2 polarization. The result using visceral WAT is shown in Fig. 3, and a similar experiment using subcutaneous WAT is shown in Fig. 4. We then examined the endogenous *Crabp1* mRNA expression pattern in the primary adipocyte differentiation process. It appeared that, endogenous *Crabp1* was initially highly expressed, then was reduced at D2 in both visceral (Fig. 3B) and subcutaneous (Fig. 4B) cells. Its expression level stayed low in mature visceral adipocytes (D10), but was slightly elevated in mature subcutaneous adipocytes (D10) (Figs. 3 and 4B). This pattern of endogenous *Crabp1* expression would suggest an early role for CRABP1 in adipocyte differentiation and, probably, its function. This pattern of *Crabp1* expression is also consistent with our previous finding using 3T3L1 adipocyte model [27]. Interestingly, fat accumulation appeared to be more efficient for CKO preadipocyte cultures, as indicated with increased lipids stained by Oil Red O (Figs. 3C and 4C). This is also consistent with the more expanded WAT in CKO mice under HFD feeding. Upon comparing macrophage polarization in the presence of adipocyte-CM, as evaluated with qPCR (Figs. 3D and 4D) and flow cytometry (Figs. 3E, F and 4E, F), we found that both CKO adipocyte-CM suppressed M2-like (marked with *Arg1* and CD206) polarization. Taken together, CKO adipocyte-CM was more effective than WT adipocyte-CM in altering M1/M2-like polarization, especially suppressing M2 polarization. This would suggest that CKO’s WAT inflammation is mostly contributed by intrinsic defects in CKO adipocytes, which disturbs the control of resident macrophage M1/M2 polarization.

### CRABP1 regulates adiponectin production via ERK1/2 signaling in adipocytes and their mitochondrial content

**Fig. 5:**
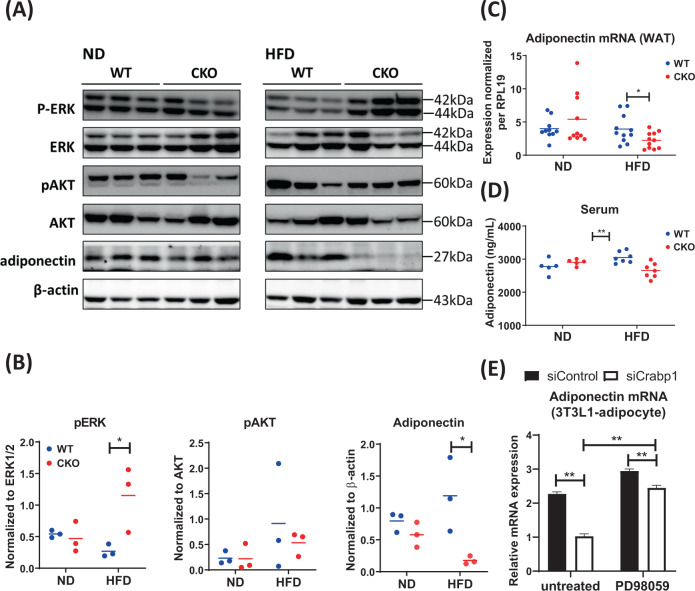
CRABP1 alters resident macrophage polarization via ERK1/2-modulated adiponectin expression in WAT. **A** Western blots of WAT harvested from ND- or HFD-fed WT and CKO mice for pAKT, pERK and adiponectin. **B** Western blot quantification of pERK, pAKT and adiponectin levels. Total ERK1/2, total AKT and *β-actin* were used as loading controls. *n* = 3. **C** qPCR of adiponectin mRNA in WAT. *n* = 10–11. **D** Adiponectin serum levels were detected by ELISA. *n* = 5. **E** qPCR of adiponectin mRNA levels. Data were collected from 3T3L1-differentiated adipocytes with or without silencing *Crabp1* on indicated days as described in the methods. Adipocytes were treated with 25 μM PD98059 for 24 h. Error bars are shown as means ± SEM. Student’s *t* test, *p < 0.05, **p < 0.01.

We previously found that CRABP1 could negatively modulate ERK1/2 signaling in 3T3L1-adipocytes [28]. ERK1/2 signaling is important in adipocytes since it is a negative regulator of adiponectin production; and adiponectin is anti-inflammatory, by regulating macrophage polarization [5, 9]. We thus compared endogenous ERK1/2 activation and adiponectin levels in WAT between WT and CKO mice (Fig. 5A). For a control, another upstream regulator of adiponectin, AKT (a positive regulator) was also examined. Clearly, under HFD, ERK1/2 activation significantly increased, without apparent increase in its protein levels in CKO’s WAT, whereas AKT activation did not change significantly. To confirm this, AKT activation in WAT was further determined by examining four additional animals (Supplementary Fig. S3A). By combining these results (Fig. 5A and Supplementary Fig. S3A), it is confirmed that AKT activation did not change significantly in CKO adipocytes (Supplementary Fig. S3B). Importantly, endogenous adiponectin was almost undetectable in CKO’s WAT, consistent with the notion that ERK1/2 activity, an inhibitor of adiponectin production, was elevated in CKO adipocytes due to the deletion of *Crabp1* which negatively regulated ERK signaling. Figure 5B shows the quantified results of these experiments. We further compared adiponectin mRNA in WAT and serum adiponectin levels (Fig. 5C, D). These data confirmed that ERK1/2 over-activation in CKO adipocytes caused a reduction in adiponectin production, resulting in lowered serum adiponectin level.

To further validate the functional significance of CRABP1, as well as the involvement of ERK1/2, in adiponectin production, we employed the established 3T3L1 in vitro adipocyte differentiation model. We silenced *Crabp1* expression in 3T3L1 preadipocyte cultures, which were then induced for adipocyte differentiation, with or without the addition of ERK inhibitor (to block ERK activation due to the absence of CRABP1). The expression of adiponectin was then monitored. As shown in Fig. 5E, silencing *Crabp1* indeed decreased the expression of adiponectin mRNA, which was rescued by the ERK inhibitor PD98059. These in vivo and in vitro data show that CRABP1 negatively modulates ERK activation in adipocytes, which contributes to maintenance of proper adiponectin expression.

**Fig. 6:**
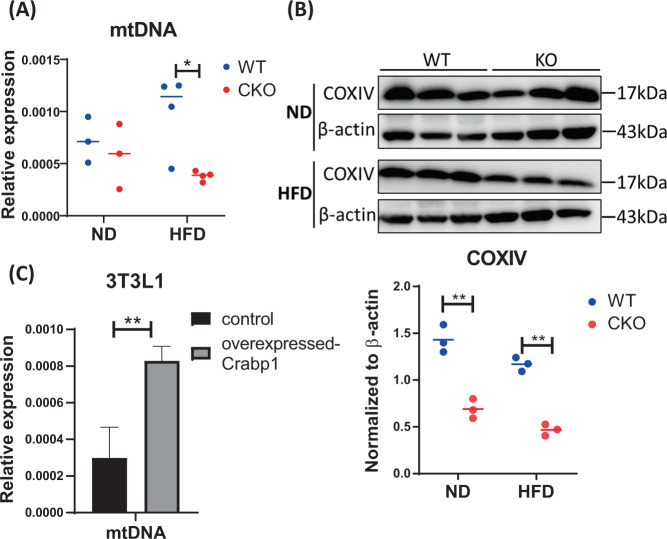
Mitochondrial defects in WAT of CKO mice. **A** qPCR to determine mitochondrial DNA contents (mtDNA). **B** upper panel Western blots of COXIV. **B** bottom panel Western blot quantification of COXIV. Total *β-actin* levels were used as loading controls. Results were obtained from two independent experiments. **C** CRABP1 is beneficial for increasing mitochondrial content. 3T3L1 cells were transfected with flag-CRABP1 and empty vector control. mtDNA was assessed via COll expression and normalized to *β-actin*. Error bars shown are means ± SEM. Student’s *t* test, *p < 0.05, **p < 0.01.

It is also known that mitochondrial homeostasis is important in maintaining healthy adipocytes and their function including adiponectin production/secretion [31, 32]. We thus also compared WAT’s mitochondrial DNA (mtDNA) content between WT and CKO. Interestingly, there was a significant reduction in mitochondrial DNA content (Fig. 6A) and protein COXIV level (Fig. 6B) in CKO, as compared to WT mice, under HFD feeding. Consistently, in the 3T3L1 model, elevating CRABP1 level could increase mtDNA content (Fig. 6C). Therefore, CRABP1 in adipocytes also affects their mitochondrion homeostasis, which could also contribute to the regulation of adipocyte health and its function such as adiponectin production/secretion.

Altogether, our data show that CRABP1 is beneficial in maintaining the health and function of adipocytes, which would protect against the development of obesity-related WAT inflammation, especially under an HFD feeding. In adipocytes, CRABP1 contributes to the maintenance of mitochondrial homeostasis and proper adiponectin production, which involves ERK1/2 signaling. Without CRABP1, adipocytes are defected, with a lowered mitochondrial content and reduced adiponectin production. This would contribute to unhealthy polarization of resident macrophages, with preferential M1 polarization, that then enhances WAT inflammation.

## Discussion

This study reports, for the first time, that CRABP1 protects mice from HFD-induced inflammation by, partially, regulating and maintaining adipocyte differentiation and function thereby modulating WAT’s resident macrophage polarization and inflammation. Mechanistically, CRABP1, in adipocytes, contributes to proper adiponectin production and mitochondrial homeostasis, which is important in enhancing resident macrophage M2-like (CD206^+^) polarization and adipose inflammation. Further, the effect of CRABP1 in adipocytes depends on its activity to negatively regulate ERK1/2 over-activation (model, Supplementary Fig. S4). This study illustrates a new mechanism controlling adipose tissue inflammation by the action of CRABP1 that prevents over-activation of ERK1/2 to ensure the health and function of adipocytes, which dampens resident macrophages’ inflammatory response by favoring M2-like and inhibiting M1-like polarization.

Vitamin A and RA play important roles in obesity by regulating adipogenesis and browning of WAT. For example, pharmacological RA has been reported to inhibit adipogenesis by activating Wnt signaling [33] and PPARγ [34], or promoting the browning of WAT by inducing beige adipose progenitors [35] via RA receptors (RARs), providing a beneficial effect in obesity prevention. Regarding the action of RA, classical studies have shown CRABP1 could trap or channel RA to cytochrome p450 for further metabolism [36] and RA could activate ERK expression via its genomic action [37, 38], thus CRABP1 plays an inhibitory role in ERK expression by the genomic action of RA. However, this requires further studies to verify if RA availability to the nucleus can be reduced by CRABP1 expression in a physiologically relevant context. Our recent studies have revealed that CRABP1-RA also dampens ERK activation in the cytoplasm, mediating the non-canonical (non RAR-dependent) activity of RA. Thus, the overall action of CRABP1 is to suppress/dampen ERK signaling. This is supported by the results of significantly enhanced ERK activity in CKO, as well as changes in downstream targets of ERK, such as reducing adiponectin in adipocytes. Of interest is a lack of apparent increase in ERK protein levels in CKO (Fig. 5A), which would be predicted from CRABP1’s suppression of the genomic effect of RA (model Supplementary Fig. S4). This would suggest a more potent effect of CRABP1 in modulating ERK activity, but not ERK expression, in the context of whole animals. Regarding the non-canonical activity of RA, studies have also reported novel effects such as dendritic translation [39, 40], and apoptosis [41]. To this end, more studies are needed to carefully study these novel findings in various physiological conditions.

ERK1/2 signaling pathway is one of CRABP1 targets [42]. ERK1/2 regulates numerous cellular processes in adipocytes, and its activity has to be controlled to maintain adipocyte health and function [17–19]. This current study first detected defect in adiponectin production in CKO adipocytes associated with the obese and inflammatory phenotype of CKO mice. In adipocytes, ERK activity is also required for important transcription factor expression/function [19, 43] that are essential for adipocyte gene expression [44]. We have previously documented that CRABP1 could also promote CEBPβ activity in vitro. Whether and how other adipocyte transcription factors, such as SREBPs are also affected by the absence of CRABP1 remains to be carefully determined.

Interestingly, the significant increase in the M1/M2 ratio of CKO’s WAT, indicative of a hyper-inflamed state, is not initiated from cell-autonomous changes in, or the intrinsic property of, WAT’s resident macrophages. Rather, it is caused by defects in CKO adipocytes. Most dramatic CKO adipocyte defects include over-activated ERK1/2 signaling and dramatically reduced mitochondrial content, associated with significantly reduced adiponectin production and expression. Given that adiponectin is critical to the maintenance of immune and metabolic homeostasis, it is of no surprise that CKO mice are more vulnerable to the development of obesity and inflammation, especially under HFD feeding. Interestingly, we examined mRNA expression of five chemokine genes, and the results suggested that those related to immune cell infiltration might not be affected by CRABP1. However, it remains to be further examined whether protein expression of these genes was affected. This will require further intensive studies.

We compared the lymphoid cell populations between CKO and WT mice and found that the percentages of B and T cells were similar between WT and CKO mice, indicating that deleting *Crabp1* probably had little effect on the development/distribution of these immune cells. Adaptive immunity is also important in obesity-induced adipose tissue inflammation [45, 46]. Since we have not rigorously examined various subpopulations of B and T cells, it remains to be seen whether the adaptive immunity of CKO mice is somewhat impaired. As to the macrophage lineage, our results showed that CKO macrophages had a preferentially M1-like phenotype, but their surface MHCII and CD86 markers were lower (Fig. 2A). This could be due to certain compensation mechanism for the increased M1/M2 ratio. Also, *Crabp1* deficiency may lead to macrophages defects in WAT. To this end, studies have indicated that macrophage can be activated by Toll-like receptors (TLRs) and IFNγ to increase the expression of their activation markers, such as CD80 and CD86 [47], and TLRs pathways can contribute to the development of obesity-associated inflammation [48]. It remains to be examined if CRABP1 plays a role in regulating inflammatory response of TLRs. In addition, MHCII presents antigen to lymphocytes to activate adaptive immune responses [49]. Thus, it remains to be examined if CKO macrophages have any defect in adaptive immune activation. While the most significant change in CKO immune cells is the apparently impaired regulation of innate immunity, at least in controlling HFD-induced local WAT inflammation (which is attributable to inadequate adiponectin and preferential resident M1-like macrophage polarization), future studies are needed to carefully examine other immune responses.

The finding that *Crabp1* expression is important to ensure adipocyte health and function is consistent with the expression pattern of *Crabp1* in this current study and our previous studies. Both our earlier in vitro (3T3L1-adipocyte differentiation model) [27] and current ex vivo (primary adipocyte differentiation from mouse adipose tissue, Figs. 3 and 4**)** studies have revealed that *Crabp1* is highly expressed in preadipocytes, downregulated in later stages of adipocyte differentiation. Presumably, CRABP1 plays a role in early adipocyte differentiation, and, possibly, also in maintaining the health/function of differentiated adipocytes. This is supported by the new finding here that, CRABP1 is important for adipocyte function (such as adiponectin production/secretion) and adipocyte health (such as mitochondrial homeostasis).

Mitochondria are important for maintaining metabolic homeostasis in numerous cell types including adipocytes. Mitochondrial dysfunction in adipocytes is associated with insulin resistance and tissue inflammation [10]. The significant reduction in mitochondria of CKO adipocytes would indicate their poor health, due to the absence of CRABP1. Since mitochondrial function is also important for adiponectin synthesis [32], this damage in CKO adipocytes could also contribute to the dramatic reduction in adiponectin production/secretion. To this end, ERK1/2 pathway also contributes to mitochondrial homeostasis by regulating PGC-1α, a key regulator of mitochondria [50]. This would suggest that dysregulation in ERK1/2, one of CRABP1’s targets, could also contribute to mitochondria damage in CKO adipocytes. It would be interesting to examine whether other mitochondria-related genes/processes are affected in CKO cells. For instance, ROS plays an important role in mitochondrial homeostasis in pathogenesis of obesity [10–12]. This will be an important direction in future studies.

Finally, we suspected a correlation of CRABP1 with human diseases associated with obesity and/or inflammation. Through mining human patient data sets, we found patients of inflammatory diseases with downregulated *CRABP1* expression (Supplementary Table 1), including multiple sclerosis [51, 52], Crohn’s Disease [53, 54], Psoriasis [55, 56], and HIV-therapy induced metabolic syndrome [57], etc. Importantly, patients of these diseases all have significantly reduced *CRABP1* expression and are associated with abnormality in adipose tissue/obesity. These patient data further support the potential link between abnormal expression of CRABP1 and defective immunity.

## Supplementary information

Supplement

## Data Availability

The data generated during this study are available upon reasonable request from the corresponding author.
