# Supplementary material for: Modulation of adipose inflammation by cellular retinoic acid-binding protein 1
Source: Int J Obes (Lond). 2022 Jul 6;46(10):1759–69. doi: 10.1038/s41366-022-01175-3 (PMC9492549; doi:10.1038/s41366-022-01175-3)
Supplement: Supplementary file 1 — Supplement [file 41366_2022_1175_MOESM1_ESM.docx]

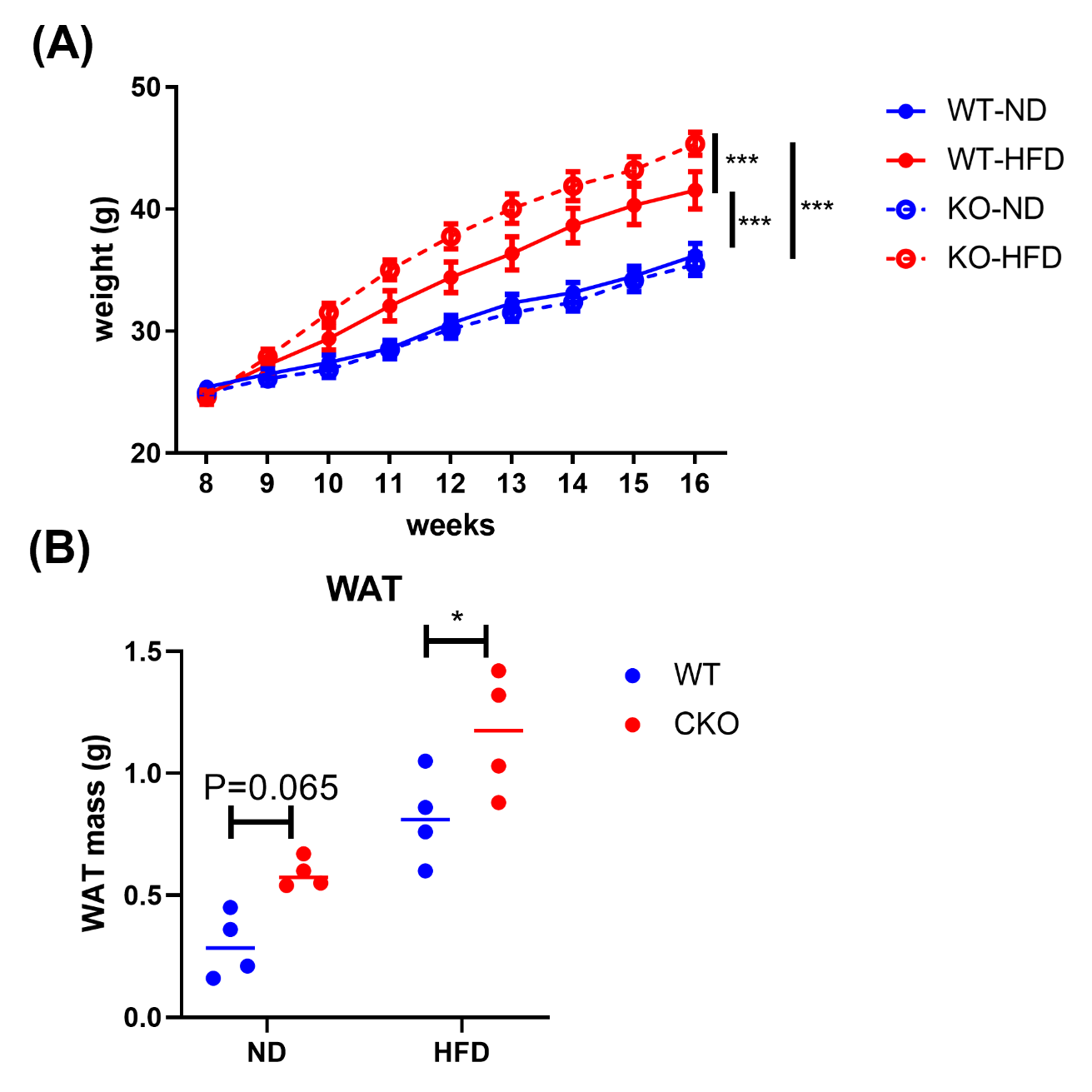


**Figure S1. CKO mice gained more weight under HFD feeding.**

(A) Average body weight of WT and CKO mice fed a ND and HFD for 8 weeks; n=11 in ND and HFD groups. Results from four independent experiments. (B) Average visceral WAT (vWAT) tissue mass (left lobe) of ND- or HFD-fed WT and CKO mice. Experiments for statistical analysis using two-way ANOVA, *p<0.05, ***p<0.01


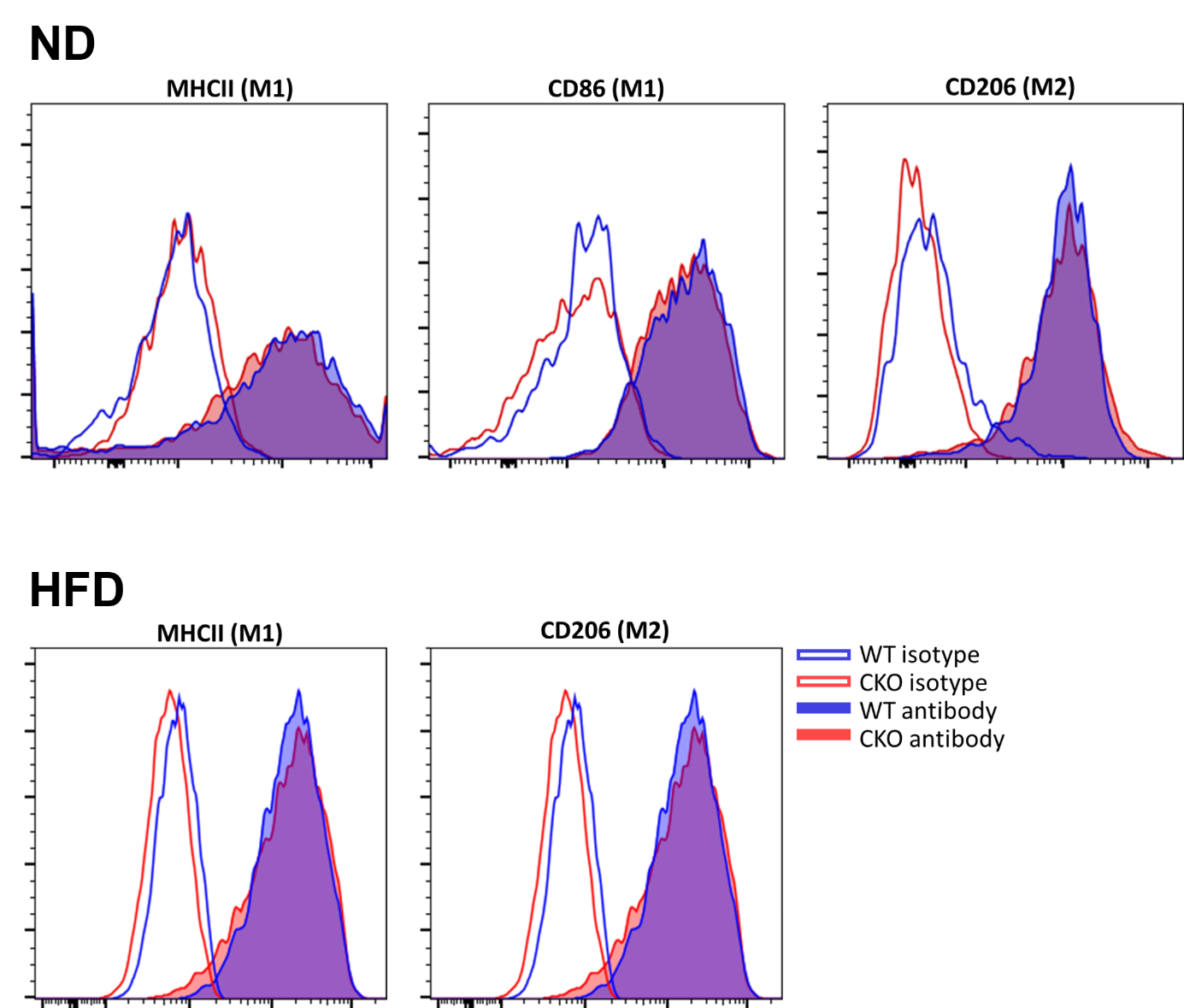


**Figure S2.** **CRABP1 deficiency does not affect the macrophage polarization in BMDM.**

BMDM from WT and CKO mice under ND or HFD feeding were differentiated into M1 via LPS and IFNγ, and into M2 via IL-4. The expression of CD86, CD206, and MHCII were examined by flow cytometry. Results shown are representative of two independent experiments.


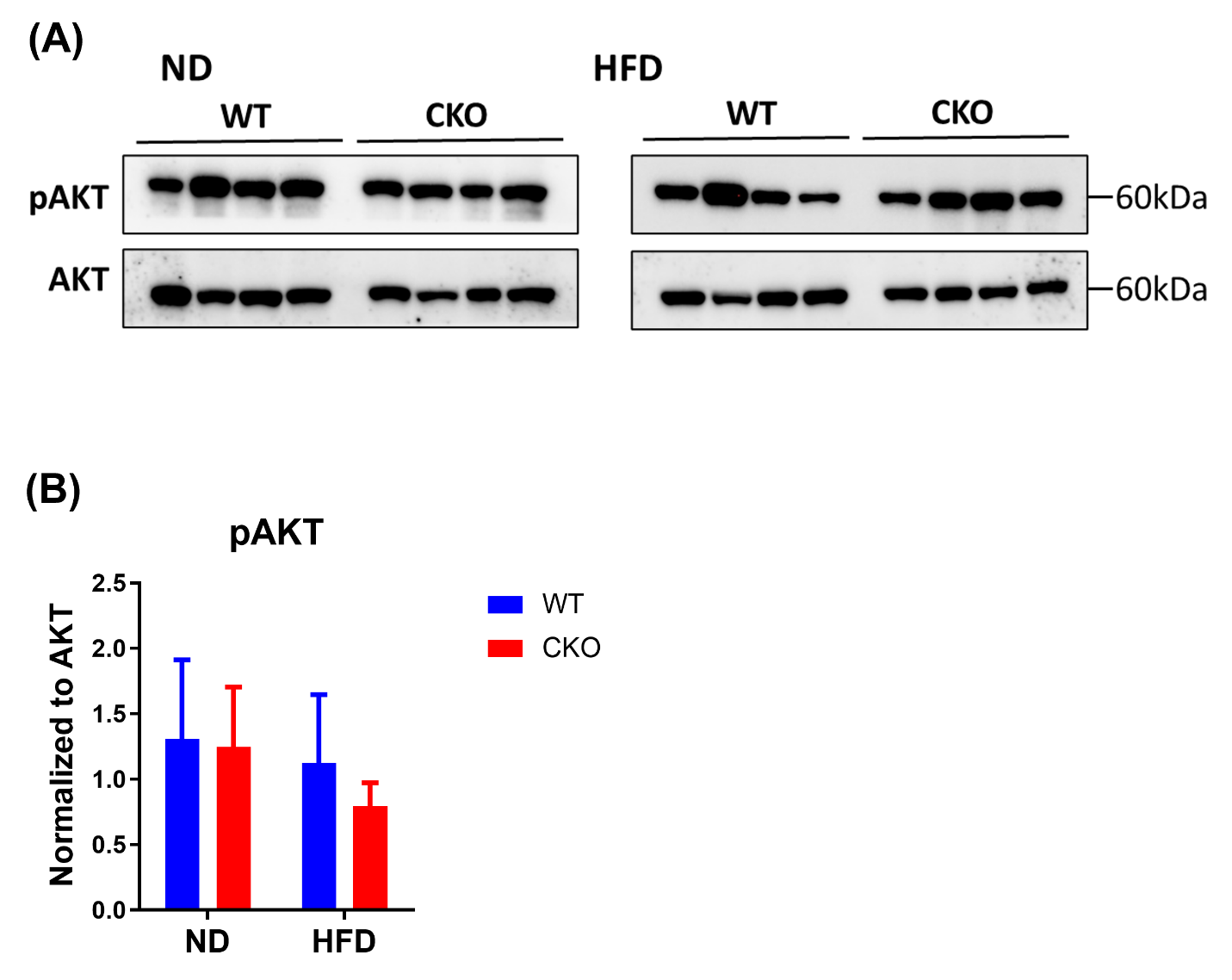


**Figure S3. CRABP1 regulates adiponectin production via AKT-independent pathway.**

(A) Western blots of WAT harvested from additional ND- or HFD-fed WT and CKO mice for analyzing pAKT. (B) Quantification of changes in pAKT by combining results from Figure 5A and Figure S3. Total AKT was used as loading control. n=7. The results show no statistically significant changes between WT and CKO in pAKT. Error bars are shown as means ± SEM.

**
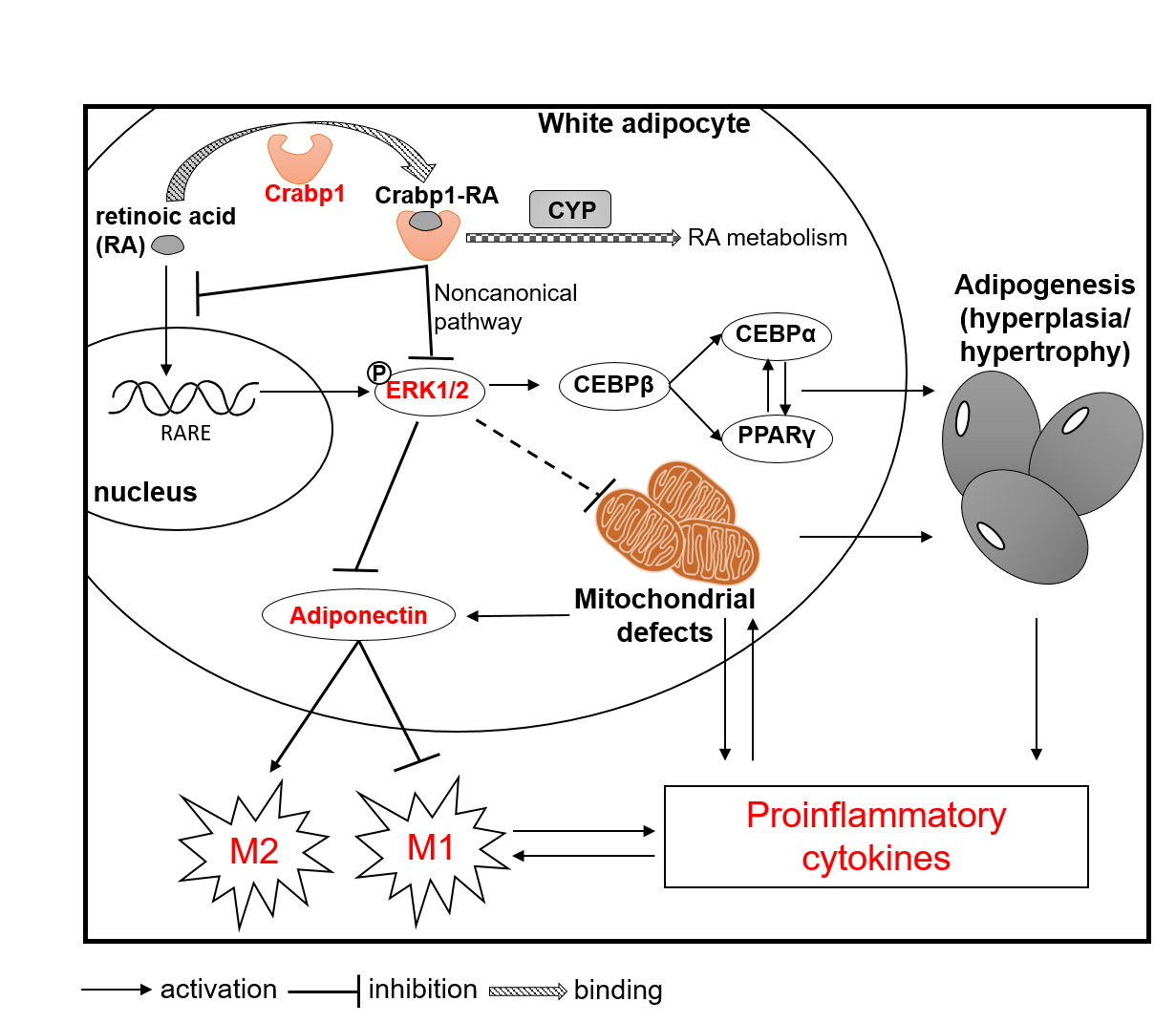
**

**Figure S4. A proposed model for the effects of CRABP1 in modulating innate immunity via regulating M1/M2 macrophage polarization.**

The classical genomic action of RA upregulates ERK1/2 expression, and CRABP1 is proposed to trap or channel RA for further metabolism, thus it inhibits RA-activated ERK1/2 expression. For the non-canonical (non RAR-dependent and cytosolic) action of RA, CRABP1-RA directly dampens activation of ERK1/2 in the cytoplasm. Thus, the overall action of CRABP1 would be to suppress ERK1/2 signaling pathway. Consequentially, ERK1/2 targets, such as adiponectin, mitochondria and CEBPβ, etc., can all be affected. Pertinent to the regulation of inflammation, adiponectin is known to inhibit M1, and promote M2, polarization, contributing to anti-inflammation. CKO mice have enhanced ERK1/2 activities, thus reducing adiponectin level and, as a result, are prone to inflammation.

**Supplementary Table 1. *CRABP1* in human inflammatory-associated disease**

| **Diseases associated with inflammation** | ***CRABP1* Fold-Change** | **Association with obesity/adipose tissue abnormality** | **Ref.** |
| --- | --- | --- | --- |
| Multiple Sclerosis (MS) | 0.53 fold-decrease in gray matter | Obesity as a risk factor for MS development and progression | [51, 52] |
| Crohn’s Disease | 0.35 fold-decrease in intestinal tissue (Ileum) | Increased adipose hypertrophy, “Fat-wrapping”, at the intestinal lining | [53, 54] |
| Psoriasis | 0.12 fold-decrease in skin | Obesity as a risk factor for psoriasis development and progression | [55, 56] |
| HIV-Therapy induced lipid dystrophy and metabolic syndrome | Inhibited Function | Disturbed adipose distribution | [57] |

**Supplemental Experimental Methods and Materials:**

**Bone marrow-derived macrophages (BMDM) and M1/M2 polarization**

Bone marrow was harvested from the femurs and tibias of 8-week-old mice. Red blood cells were removed with ACK buffer. Single-cell suspension of bone marrow was cultured in completed DMEM (Thermo Fisher Scientific #11960044) containing, 100 U/mL penicillin, 100 mg/mL streptomycin, 2 mM L-glutamine and 10 % heat-inactivated FBS at 37˚°C for 1 hr. Non-adherent cells were then collected and cells were re-plated in completed DMEM containing 10% L929 condition medium. Fresh medium was replaced after three days of culture. On day 6, BMDM were harvested for experiments. For M1 and M2 polarization, BMDM (M0) were differentiated to M1 by 100 ng/mL LPS and 50 ng/mL IFNγ (Biolegend #575304), and M2 by 10 ng/mL IL-4 (Biolegend #574302). After 24 hours, cells were detached using 5 mM EDTA in PBS and analyzed by flow cytometry.

**Quantitative RT-PCR**

RNA was extracted by TRIzol Reagent (Ambion). RNA concentration was measured with the NanoDrop and cDNA was synthesized by High-Capacity cDNA Reverse Transcription Kit (Applied Biosystems™ #4368814). Quantitative RT-PCR was performed using the SYBR™ Green PCR Master Mix (ThermoFisher, # K0253). Real-time RT-PCR was conducted on Mx3000P QPCR Systems (Agilent). Primers for *Adipoq* F, 5’ AGATGGCACTCCTGGAGAGA 3’; *Adipoq* R, 5’ ACATAAGCGGCTTCTCCAGG 3’; *Crabp1* F, 5’ CGGAGATCAACTTCAAGGTCGG 3’; *Crabp1* F, 5’ CCCTCAAGAAGTGTCTGTGTGC 3’; *iNOS* F, 5’ GAGACAGGGAAGTCTGAAGCA 3’; *iNOS* R, 5’ CCAGCAGTAGTTGCTCCTCTT 3’; *Arg1* F, 5’ CATTGGCTTGCGAGACGTAGAC 3’; *Arg1* R, 5’ GCTGAAGGTCTCTTCCATCACC 3’; *RPL19* F, 5’ TCATCCGCAAGCCTGTGACT 3’; *RPL19* R, 5’ CTTCTCAGGCATCCGAGCAT 3’; *GAPDH* F, 5’ ACTCCACTCACGGCAAATTC 3’; *GAPDH* R, 5’ TCTCCATGGTGGTGAAGACA 3’; *β-actin* F, 5’ TGGCCTTAGGGTTCAGGGGG 3’; *β-actin* R, 5’ GTGGGCCGCTCTAGGCACCA 3’.

**Oil Red O staining**

Oil Red O stock solution (0.5% Oil Red O dissolved in isopropanol) was dissolved to 0.3% working solution in water. Cells were fixed with 10% formaldehyde for 10 min. at room temperature. Washed, cells were stained with Oil Red O working solution for 15 minutes.

**ELISA**

Whole blood was collected via cardiac puncture and allowed to coagulate for 30 min at room temperature. Samples were then centrifuged at 10,000g at 4°C for 20 min., and the supernatants were collected as serum samples. Serum level of Adiponectin was measured with Mouse Adiponectin/Acrp30 Quantikine ELISA Kit (R&D #MPR300) according to manufacturer’s instructions.

**Western blotting**

Cells were lysed in lysis buffer (50 mM HEPES, 0.1 mM EGTA, 0.1 mM EDTA, 120 mM NaCl, 0.5% sodium deoxycholate, 0.1% SDS, 1 mM NaF, 1 mg/ml NaVO_4_, 1 mM PMSF and 1X Protease Inhibitor Cocktail (ThermoFisher #78439)) and incubated on ice for 20 mins and then centrifuged at 14000g, 4°C for 20 min. Protein quantification of whole cell lysate supernatant was performed using Bradford Assay (Biorad #500-0006). Protein lysates were boiled in 4X sample buffer (Bio-Rad #1610747) for 10 min and electrophoresed on SDS polyacrylamide gels and transferred onto PVDF membranes (Millipore). The membrane was then blocked with 5% non-fat milk in 1x TBST at room temperature for 1 hr. Membranes were washed four times for 5 min with 1x TBST (0.1% Tween-20 in TBS) and incubated with primary antibodies listed in the table below with antibody buffer (0.1% Tween 20 and 2% BSA in TBS) overnight and then in secondary horseradish peroxidase-conjugated antibody (HRP; GeneTex). HRP signal was detected using Immobilon Western Chemiluminescent HRP substrate (Advansta). Images were acquired with Bio-Rad ChemiDoc Imager (Bio-Rad Laboratories) and the relative level of protein was analyzed by ImageLab software.

**Mitochondrial DNA quantification**

Cell total DNA was extracted using genomic DNA extraction kit (Qiagen Dneasy blood & tissue kit) according to manufacturer’s instructions. COII expression for mitochondrial DNA abundance analysis by SYBR™ Green PCR Master Mix (ThermoFisher, # K0253) with 50 ng of total DNA in combination of COII.F, 5’ TGAGCCATCCCTTCACTAGG 3’; COII.R, 5’ TGAGCCGCAAATTTCAGAG 3’ or *β-actin* F, 5’ TGTTCCCTTCCACAGGGTGT 3’; *β-actin* R, 5’ TCCCAGTTGGTAACAATGCCA 3’ primers ^1^. Real-time RT-PCR was conducted on Mx3000P QPCR Systems (Agilent). Relative mtDNA were measured and calculated by normalizing β-actin expression level to COII level.

**Plasmid and transfection**

For *Crabp1* overexpression, the flag-Crabp1 expression construct was prepared as described ^2^. 3T3L1 were seeded 6-cm dishes, and 2.5 µg of flag-Crabp1 or control plasmid were transfected into cells with Lipofectamine 2000 transfection reagent (Invitrogen) according to the manufacturer’s protocol. One day later, cells were treated with DMSO or 25 µM PD98059 for 24 hrs.

# **References**

1. Piantadosi CA, Suliman HB. Mitochondrial transcription factor A induction by redox activation of nuclear respiratory factor 1. *J Biol Chem* 2006; **281**(1)**:** 324-33.

2. Park SW, Persaud SD, Ogokeh S, Meyers TA, Townsend D, Wei LN. CRABP1 protects the heart from isoproterenol-induced acute and chronic remodeling. *J Endocrinol* 2018; **236**(3)**:** 151-165.

**Antibodies used in flow cytometric and western blot analysis:**

| **Antibody** | **Clone/Cat.#** | **Vendor** |
| --- | --- | --- |
| **CD45** | **QA17A26** | **biolegend** |
| **TCRb** | **H57-579** | **biolegend** |
| **CD8** | **53-6.7** | **biolegend** |
| **CD4** | **GK1.5** | **biolegend** |
| **B220** | **RA3-6B2** | **biolegend** |
| **F4/80** | **BM8** | **biolegend** |
| **CD11b** | **M1/70** | **biolegend** |
| **Gr-1** | **RB6-8C5** | **biolegend** |
| **CD11c** | **N418** | **biolegend** |
| **MHCII (I-A/I-E)** | **M5/114.15.2** | **biolegend** |
| **CD206** | **MMR** | **biolegend** |
| **CD86** | **GL-1** | **biolegend** |
| **Rat IgG2b, κ** | **RTK4530** | **biolegend** |
| **Rat IgG2a, κ** | **RTK2758** | **biolegend** |
| **Adiponectin** | **#2789** | **Cell signaling** |
| **β-actin** | **# sc-47778** | **Santa Cruz** |
| **Phospho-p44/42 MAPK (Erk1/2)** | **#9101** | **Cell signaling** |
| **p44/42 MAPK (Erk1/2)** | **#9102** | **Cell signaling** |
| **Phospho-Akt (Ser473)** | **#4060** | **Cell signaling** |
| **Akt** | **#9272** | **Cell signaling** |
| **COXIV** | **#ab16056** | **Abcam** |
